# A Rapid Label-Free Disposable Electrochemical Salivary Point-of-Care Sensor for SARS-CoV-2 Detection and Quantification

**DOI:** 10.3390/s23010433

**Published:** 2022-12-30

**Authors:** Nadia Farsaeivahid, Christian Grenier, Sheyda Nazarian, Ming L. Wang

**Affiliations:** 1 Interdisciplinary Engineering Program, Northeastern University, Boston, MA 02115, USA; 2 Civil and Environmental Engineering Department, Northeastern University, Boston, MA 02115, USA

**Keywords:** COVID-19, SARS-CoV-2 detection, saliva, biosensor, spike protein, electrochemistry, point-of-care

## Abstract

The coronavirus disease 2019 (COVID-19) pandemic has created an urgent need for accurate early diagnosis and monitoring. A label-free rapid electrochemical point-of-care (POC) biosensor for SARS-CoV-2 detection in human saliva is reported here to help address the shortcomings of traditional nucleic acid amplification methods and give a quantitative assessment of the viral load to track infection status anywhere, using disposable electrochemical sensor chips. A new chemical construct of gold nanoparticles (GNp) and thionine (Th) are immobilized on carboxylic acid functionalized carbon nanotubes (SWCNT-COOH) for high-performance biosensing. The sensor uses saliva with a one-step pretreatment and simple testing procedure as an analytical medium due to the user-friendly and non-invasive nature of its procurement from patients. The sensor has a response time of 5 min with a limit of detection (LOD) reaching 200 and 500 pM for the freely suspended spike (S) protein in phosphate buffer saline (PBS) and human saliva, respectively. The sensor’s performance was also proven for detecting a COVID-19 pseudovirus in an electrolyte solution with a LOD of 10^6^ copies/mL. The results demonstrate that the optimized POC sensor developed in this work is a promising device for the label-free electrochemical biosensing detection of SARS-CoV-2 and different species of viruses.

## 1. Introduction

In early December 2019, SARS-CoV-2, the virus that caused coronavirus disease 2019, was discovered in Wuhan and quickly spread across the world. This new virus belongs to the Coronaviridae family along with SARS-CoV and MERS-CoV, which were regional epidemics in 2003 and 2012, respectively [1,2,3,4,5]. SARS-CoV-2 itself is composed of four different proteins, spike (S), membrane (M), nucleocapsid (N), and envelope (E), with the single strand ribonucleic acid (ssRNA) encapsulated inside the virion. Due to the S glycoprotein’s critical importance to the virus for cell entry and as it decorates the outside of the virion, it is the main target of neutralizing antibodies and vaccine design [6,7]. Patients suffer from a variety of visible symptoms with onset 3–5 days after infection, but even asymptomatic or pre-symptomatic patients are susceptible to the transmission of the virus to the uninfected before being identified for home isolation or hospitalization [8]. Therefore, the ability to identify asymptomatic or pre-symptomatic carriers is one of the main challenges facing the public response to rapidly spreading pandemics. In response to this pressure, several diagnostic methods have been developed. Reverse transcriptase polymerase chain reaction (RT-PCR), which utilizes upper respiratory swabs, is the current gold standard and dominates other methods of detection in prevalence [9,10]. Despite its laudable accuracy, this method suffers from limitations that hold it back from being optimized, such as its cost to users; a turnaround time of hours to days during times of high throughput, making it unable to keep up with the pace of community transmission; and it requires trained lab technicians at a centralized location to conduct the tests [11,12,13,14,15,16].

Microfluidic-based devices provide high accuracy with a relatively simple operation method for quantifying antibodies and proteins [17]. For instance, Siqi et al. integrated a quartz crystal microbalance (QCM) with a microfluidic channel to trap micron-sized polystyrene particles simulating bacterial strains [18]. Among various microfluidic devices, paper-based microfluidics, such as lateral flow immunoassays (LFIAs), have been widely used as a diagnostic POC system [19]. LFIAs are a type of SARS-CoV-2 diagnostic POC system based on antigen-antibody (Ab-Ag) interactions which have gained significant prevalence due to their ease of use, convenience, and rapidity [20]. These colorimetric tests are less complex, cost less than RNA amplification tests, give results in a short period of time (10–30 min), and can be bought over the counter (OTC). However, the results are typically qualitative, much less sensitive than PCR tests, and are prone to false negative results, with some on the market as high as 60%, especially during the early stage of infection and given a low viral load [21,22]. As well, LFIAs require labeled secondary antibodies, adding steps and complexity [23]. An additional problem with LFIAs and PCR tests is the methods of sample collection and preparation with multiple pretesting steps, which add complexity to the user’s experience. The collection of nasopharyngeal/oropharyngeal (NP/OP) swabs has some limitations, such as the lower level of comfort with the invasiveness of the swab itself and the high risk of disease transmission to healthcare workers and others at centralized locations [24,25,26,27].

Apart from laboratory-based methods and immunoassays, electrochemical biosensor systems show promising potential as a POC and OTC method for the early detection of COVID-19 due to their selectivity and sensitivity [28,29]. Electrochemical biosensors are simple, cost-effective, and rapid, with cutting-edge nanotechnology helping to increase sensitivity and lower the LOD [30]. In recent years, electrochemical sensors have been developed to detect infectious diseases such as influenza, hepatitis B, the zika virus, and the SARS and MERS coronavirus [31].

Viral electrochemical sensors (including for SARS-CoV-2 detection) have been widely reported in the literature [32,33,34,35,36,37,38,39]. Raziq et al., reported a sensitive portable MIP-based electrochemical sensor for detecting SARS-CoV-2 antigens in 15 min with a limit of detection of 15 fM. Mahari et al., developed a novel electrochemical device to detect COVID-19 antigens in saliva in a few seconds. In these works, despite the low limit of detection and quick response, K3 [Fe(CN)6]/K4[Fe(CN)6] needs to be added to the sample as a redox couple, making the procedure unnecessarily complicated for non-expert use [40,41]. Fabili et al., reported a design of an electrochemical immunosensor to detect SARS-CoV-2 in untreated saliva with the LOD down to 19 ng/mL for the S protein. However, the testing time was 30 min, and the fabrication procedure required using secondary antibodies, which added an extra step to the testing procedure [33]. Chaibun et al., designed a rapid electrochemical biosensor for SARS-CoV-2 RNA detection in NP samples with a limit of detection of one copy/μL, using isothermal rolling circle amplification. Although it was accurate, this platform suffered from some limitations, including invasive sample collection, multiple pretesting steps, and time consumption [42].

Despite previous efforts to develop COVID-19 biosensors, a disposable biosensor test that can be easily performed by non-expert users with a single pretest step is required. In addition, it is crucial to use a non-invasive sample with a high level of comfort to minimize the chance of exposing healthcare workers to the virus. In this study, we develop a label-free electrochemical biosensor using nanomaterials to help measure the change in electron transfer characteristics of the functional chemistry at the surface of an electrode caused by the interaction between the bioreceptor (Ab) and the surface proteins of SARS-CoV-2 (Ag). This POC biosensor can detect and quantify the active SARS-CoV-2 S protein concentration in PBS in an individual’s saliva sample and can detect an S protein-carrying pseudovirus that mimics SAR-CoV-2 in Dulbecco’s Modified Eagle Medium (DMEM) with a 10% fetal bovine serum (FBS) solution without additional redox probes or labels added to the sample solution. With this electrochemical design, we are able to quantitatively assess the viral load to track the infection status using saliva with only one-step sample pretreatment and a simple testing procedure. The sensor is designed for portability and versatility and can be used at home, school, work, and even ports of entry without the need for medically trained technicians, complex testing procedures, and with no risk of exposing healthcare workers to the virus. The technology described in this paper has great potential to move out of the lab into commercial POC and OTC diagnostic spaces with a competitive advantage over existing technologies. In addition, this work constitutes a platform that is a process by which POC and OTC devices can be rapidly developed to combat emerging epi/pandemics in the future.

## 2. Theory and Design of the Drop-Cast Chemical Platform

The biosensor construction comprises SWCNT-COOH to increase the effective surface area, allow for higher loading of the relevant bioreceptor, and mediate electron transfer kinetics for a wide range of electroactive species [43,44]. GNp are added for bioreceptor immobilization and signal amplification due to their desirable electrical properties [45]. The commercially available anti-S1 protein antibody (S1-Ab) is used as a SARS-CoV-2 specific bioreceptor due to its high affinity with the spike protein [46]. As most Ab-Ag reactions are not direct electron transfers, it is often necessary to add a secondary redox probe, such as ferricyanide ((Fe (CN)_6_)^3−/4−^) or Th, to the solution whose reaction will correlate to the amount of Ab-Ag binding. These redox probes effectively occlude some of the surfaces and decrease the amount of reaction the latent redox probe undergoes, resulting in the lowering of the output current. This makes the testing process more complex for the user and introduces additional variability. Similarly, other electrochemical sensors or available POC devices on the market use a secondary Ab labeled with a redox probe that first attaches to the Ag in solution, and then the pair attaches to the electrode surface via another Ab to produce the output current [47,48,49]. This increases biosensor response time and similarly adds an additional step where the secondary Ab with a redox probe attached needs to be added to the sample before testing.

In contrast, the sensor described herein is label-free, with no solutions added to the testing sample, reducing the time and complexity. Th is embedded in precise proportions as a layer in the functional chemistry such that an extra added solution with a redox probe molecule and/or secondary Ab during the test is not needed. In this configuration, Th is already local to the surface double layer and participates in the reaction during the measurement. However, to sustain the reaction of Th at the double layer, hydrogen is required according to the reaction Equation (1), as well as additional electrolytic ions provided from the bulk to sustain the current. These ions in the bulk must migrate towards the surface to reach the reacting Th. When the virus or antigen binds to the surface, it inhibits the diffusion of ions from the bulk to the double layer, and a change is observed in the Th reaction under a square wave voltametric test (SWV).

(1) $$Thionine+2e^{-}+2H^{+}\;⇌\;Leucothionine$$

**Figure 1:**
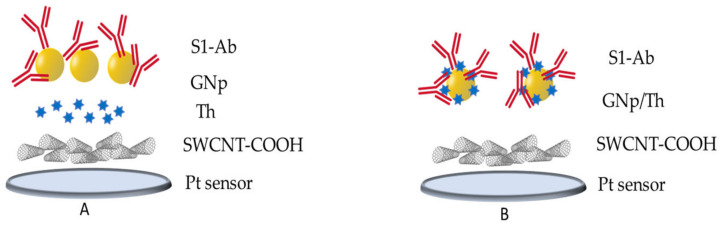
The LBL chemistry design (**A**) and hybrid LBL chemistry design (**B**).

Figure 1A represents a layer-by-layer (LBL) chemistry design that consists of (i) SWCNT-COOH, (ii) Th, (iii) GNp, and (iv) S1-Ab. During measurement using this chemistry design, the S protein in the bulk undergoes conformational binding with the Ab attached to the surface and changes the ion pathway of the Th reaction. When trying to sense larger targets such as whole virions, a modification of the chemistry is needed as the concentration of full virions in a solution is lower than the subsequent concentration of the S protein generated by viral lysis. Th, as an indicator, must be more accessible to the solution and close enough to the antibodies to detect small changes in the double layer due to Ab-Ag binding. To help with this, a hybrid design of GNp and Th was used to increase the sensitivity of the platform to the low concertation of the analyte. Figure 1B demonstrates the LBL structure of a hybrid design composed of (i) SWCNT-COOH, (ii) Th/GNp, and (iii) S1-Ab. With this hybrid design, GNp and Th provide a framework together grafted to the CNT-COOH. When the binding of an entire virion occurs on the surface, it can attach to several Abs and drastically change the interaction of Th with the analyte and the surface.

As a result, a change in the Th reaction was observed in the output signal.

## 3. Materials and Methods

SARS-CoV-2 monoclonal spike antibody and spike protein of SARS-CoV-2 were purchased from Genscript. COVID-19 spike protein pseudovirus was purchased from MyBioSource. Gold nanoparticles (GNp, 10 nm diameter), PBS (0.1 mol/L, pH 7.4), and fetal bovine serum were purchased from Corning Company. Influenza A and B antigens were purchased from Genscript. Ultrapure water was purchased from a Milli-Q plus system. Thionine acetate salt and tergitol 15-s-15 surfactant were purchased from Sigma Aldrich. Mouse IgG control antibody was from Genscript, and semiconducting COOH functionalized single-walled carbon nanotube suspension (SWCNT-COOH, diameter: 1–2 nm, length: 2–5 μm, 4000 mg/L in distilled (DI) water with ~5–7 wt.% COOH groups at the end) was from Brewer Science Company. Facilities used were in the Gorge J. Kostas Nanoscale Technology and Manufacturing Research Center, which includes a Supra 25 SEM.

### 3.1. Biosensor Fabrication Procedure

#### 3.1.1. Sensor Design

Fabrication of the disposable nano-biosensor is described in this section. The sputtered sensor chips can be made with different substrates and materials, including ceramic, aluminum, polyethylene terephthalate (PET), plastic, and glass, whereas the electrode materials are commonly provided with a range of materials such as carbon (C), platinum (Pt), gold (Au), silver (Ag), copper (Cu), and titanium (Ti). All the materials involved can be applied and cured at relatively low temperatures, which allows for an inexpensive and scalable manufacturing process.

**Figure 2:**
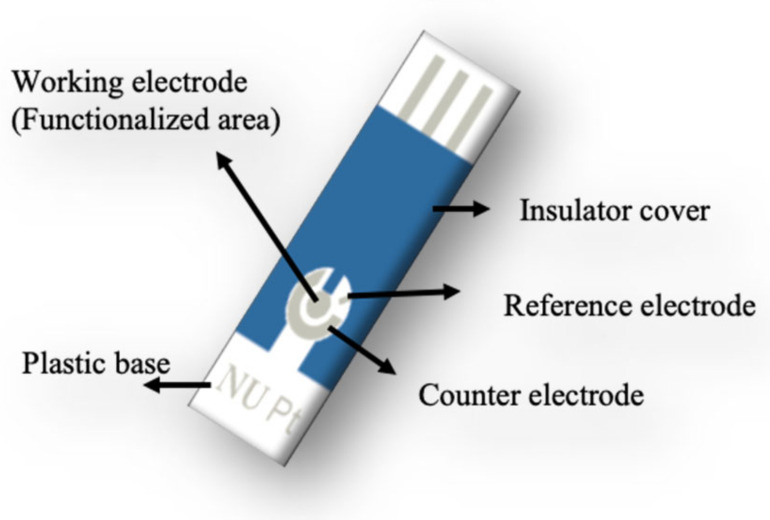
Microfabrication of sensor chip.

The bare electrochemical sensor contains at least one working electrode, a counter electrode (CE), and a reference electrode (RE), in a planar configuration, as shown in Figure 2. The WE, with a surface area of 12 mm^2^, marks out the area where the relevant reaction occurs and is the center of the dropped sample. Pt is used as the working electrode base material for its chemical inertness and favorable electrochemical properties such that the surface will not corrode or introduce contamination with the potentials applied to the cell [50,51,52]. The 3-electrode configuration also comprises a RE and CE that set the electrochemical potential and allow current to flow to sustain the reaction, respectively. All the Pt sputtered sensor chips were designed with unique specifications by Zensor R&D based in Taiwan.

#### 3.1.2. Fabrication of Drop-Cast Chemistry

**Figure 3:**
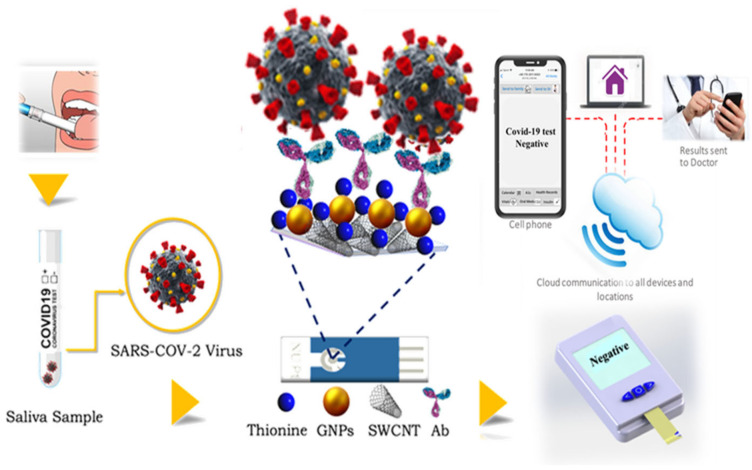
Salivary sensing system for COVID-19 detection.

The platinum electrode is washed with a DI water rinse and dried with compressed air. The first layer of the sensor is SWCNT-COOH, which is first prepared using ultra-sonification for uniform dispersion. In brief, 1 mg/mL of functionalized SWCNT-COOH powder is sonicated in DI water containing tergitol for 40 min, then 1 μL of the solution is drop-cast on the WE surface, and dried in a desiccator under a specific humidity for 20 min. Th solution is then dissolved in water and incubated with GNp (10 nm) at room temperature and in dark conditions for some minutes, and the total mixed solution is cast on the WE as the final layer. The immobilization of Ab on top of the functional chemistry is achieved by dispersing 100 μg/mL of S1-Ab in 0.1 M PBS, then dropping 2 μL of Ab solution on the GNp+Th/SWCNT-COOH and putting it in a desiccator to dry (10–15%). After functionalization is completed, the modified sensor is placed in a vacuum-sealed dark container and stored at 4 °C for later use. The salivary sensing system can be seen in Figure 3. For measurement, the sensors are inserted into a Palmsens4 lab potentiostat and incubated with a sample solution for a short time before running the test.

#### 3.1.3. Simplified Saliva Sampling Procedures for Test Subject

The sampling challenges for medical POC diagnostics using lab-on-a-chip devices would demand more efforts with innovative designs coordinating with the biosensor’s development. In the healthcare sector, there is a growing need for non-invasive, miniaturized, rapid, easy-to-use, and portable devices capable of being applied in clinical settings or for self-assessment at home. For this study, saliva is collected from healthy volunteers in the age range of 20–30 years.

The following procedure is given to adequately perform the test on subjects:

1. Rinse the mouth with water 15 min before collection.
2. Place the collector device with filter membrane and absorbent pad in the mouth and masticate until saturated with saliva (30–60 s).
3. Place the saliva collector into the syringe containing the specific hydrophilic and low-binding protein filter membrane with a defined weight without touching it.
4. Squeeze the plunger into the syringe to pass the sample through the filter and to the sterile tube.
5. Prepare different concentrations of S protein solution using the patient’s filtered saliva as a baseline solution.
6. Drop saliva solution containing a specific concentration of S protein on WE, and wait for 5 min.
7. After 5 min, perform square wave voltammetry (SWV) in a relevant range.
8. Dispose of the sensor.

#### 3.1.4. Experimental Procedure and Analysis

**Figure 4:**
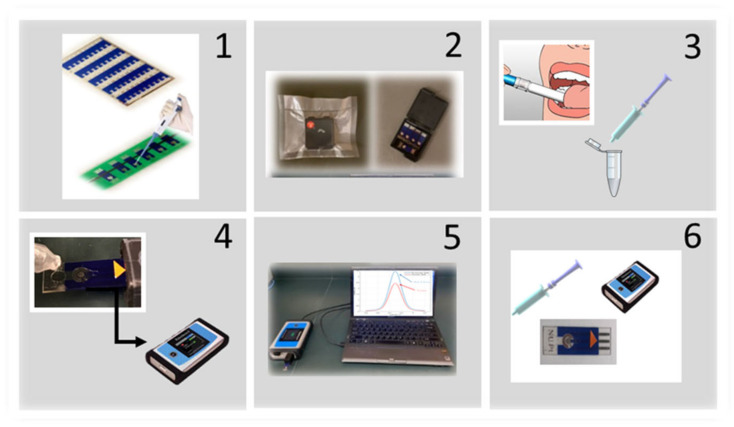
(**1**) Demonstrating sensors before cutting and fabrication, and sensors are separated and fixed on a tray during fabrication. (**2**) Demonstrating sensors’ storage gel box and how they are vacuum-packed during storage. (**3**) Insert the absorbent pad into the mouth and masticate for ~30 s. Insert the wetted absorbent pad into the syringe barrel, and squeeze the absorbent pad using the plunger. (**4**) Test setup; a portable potentiostat connected to a laptop to perform the electrochemical tests. (**5**) Positive/negative results, and the viral load will be shown on the screen. (**6**) An overview of the whole testing package.

Electrochemical measurements were carried out using the PalmSens4 potentiostat (Palm Instrument, GA Houten, The Netherlands) connected to a laptop, and testing and data analysis were performed on pre-installed software (Palm Instrument, GA Houten, The Netherlands). SWV was carried out to detect spike protein and SARS-CoV-2 pseudovirus. To initiate the measurement, the sensor was connected to the potentiostat through the sensor connector provided by Palmsens. Then, 20 μL of the solution was dropped onto the electrode area to cover all three electrodes of the sensor. After the incubation time, the electrochemical test was started, and the output signal was recorded. To establish a baseline, a control experiment was conducted using a baseline (blank) solution. Thereafter, measurements were repeated using an analyte solution. The antibody–antigen binding was analyzed by monitoring the shift of current (Δi) from the baseline solution as:

(2) $$\Delta i=i_{\mathrm{pB}}-i_{\mathrm{pS}}$$

where i_pB_ is the current of the baseline solution, and i_pS_ is the current of the analyte solution. It should be noted that all the experiments were repeated three times to ensure the reproducibility of the results. The overview of the sensor preparation and performance is represented in Figure 4.

## 4. Results and Discussion

### 4.1. Characterization of the Biosensor

#### 4.1.1. Scanning Electron Microscopy (SEM)

**Figure 5:**
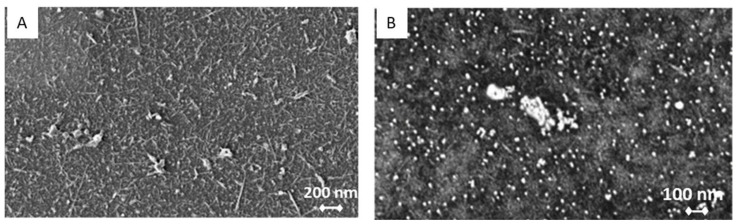
Scanning electron microscope (SEM) image of (**A**) one layer single-walled SWCNT-COOH and (**B**) SWCNT-COOH/GNp + Th.

SEM is used to obtain information about the sensor’s surface topography and composition. A single layer of SWCNT-COOH is uniformly dispersed and distributed onto the electrode surface (Figure 5A). GNp and Th are uniformly distributed with a minor amount of aggregation. SWCNT can be clearly observed underneath the GNp and Th layer (Figure 5B).

#### 4.1.2. Electrochemical Characterization

**Figure 6:**
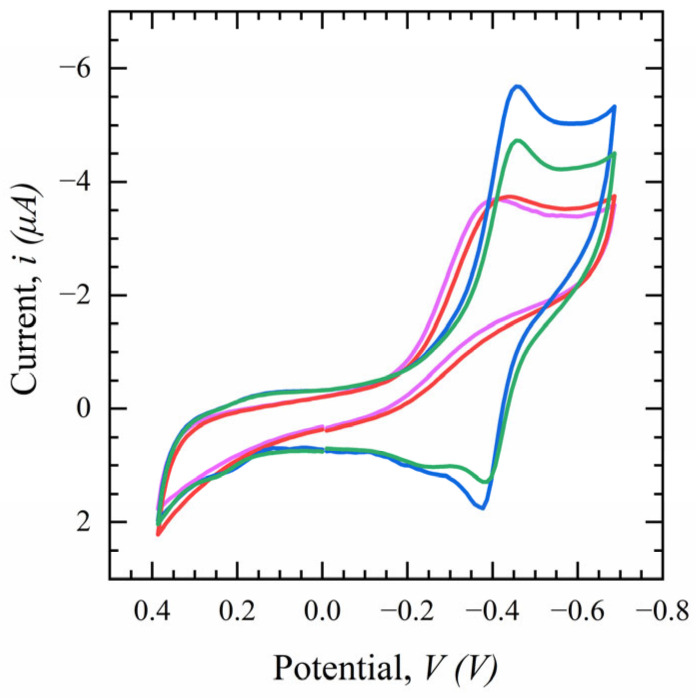
Cyclic voltammetry (CV) of the modified electrode in the presence of PBS 0.1 M. (Red: bare sensor, purple: CNT-COOH, blue: CNT-COOH/Th + GNp, green: CNT-COOH/Th + GNP/Ab), (*n* = 3).

Figure 6 shows the cyclic voltammetric behavior at different stages of fabrication at a scan rate of 0.1 V/s in a potential range of 0.4 to −0.7 V in 0.1 M PBS. It is clear from the figure that the current of the modified electrode increases after the addition of the SWCNT and Th + GNp layers due to an increase in the effective surface area compared to the bare electrode. Moreover, there is an extra oxidation peak at −0.4 V, which is attributed to the excellent electron transfer kinetics of Th. After the deposition of the S1-Ab on the modified electrode, the peak currents decrease due to the final layer of Ab acting as an electron transfer blocking agent which inhibits the bulk reaction at the surface.

### 4.2. Biosensor Optimization

**Figure 7:**
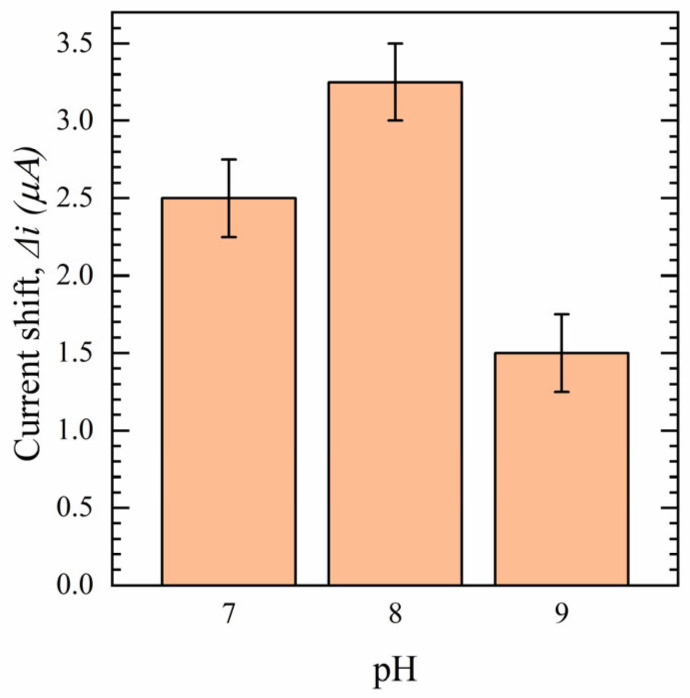
Effect of pH on sensor performance in 100 nM S protein in 0.1 M PBS (*n* = 3).

The effect of several parameters, such as pH, Ab concentration, and equilibrium (incubation) time on Δi, are investigated to achieve the highest fidelity between the concentrations of the analyte. pH can systematically control the surface charge distribution on an Ab and affect the Ab direction during immobilization on the surface [53,54]. Sensors were fabricated using S1-Ab solutions in different ranges of pH from 7 to 9. The experiments were conducted using 100 nM S protein solution in 0.1 M PBS. The results are shown in Figure 7. As can be seen, the solution with pH 8 shows a maximum Δi, indicating that keeping the pH in this range causes a change in the Ab surface charge. At pH 8, Ab would be expected to carry a net positive charge which facilitates electrostatic attraction with the negative charge of the surface, resulting in increasing the surface loading of S1-Abs. However, a significant drop in Δi is observed at pH = 9, which is related to Ab denaturation.

**Figure 8:**
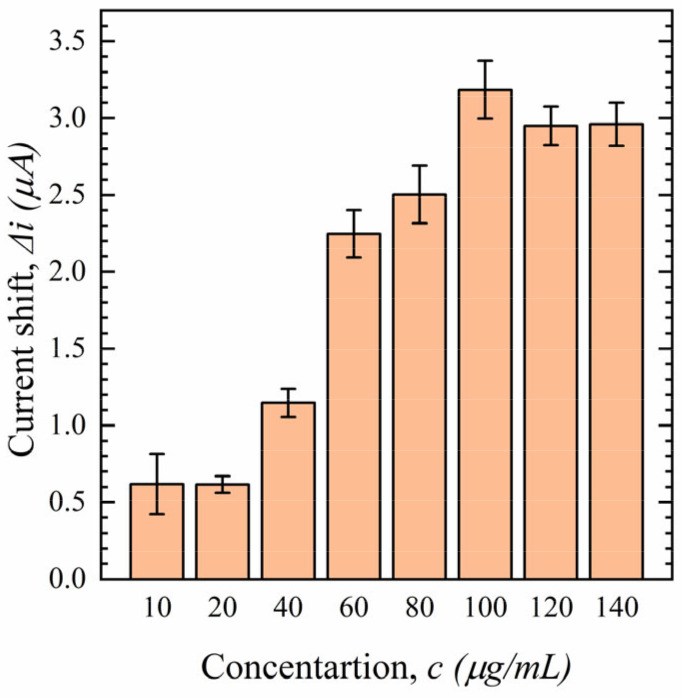
Effect of different S1-Ab concentrations on sensor performance in 100 nM S protein in 0.1 M PBS (*n* = 3).

To investigate the effect of Ab concentration, the electrode is incubated with different concentrations of S1-Ab ranging from 10 to 140 µg/mL during the drop-cast phase. Experiments were performed using 100 nM S protein, and the results are shown in Figure 8. As the S1-Ab concentration increases, the Δi increases. It reaches its maximum value at a concentration of 100 µg/mL and becomes nearly constant at higher concentrations (120 and 140 µg/mL) as the electrode surface becomes saturated. Therefore, a concentration of 100 µg/mL has been chosen as the Ab concentration for further experiments.

**Figure 9:**
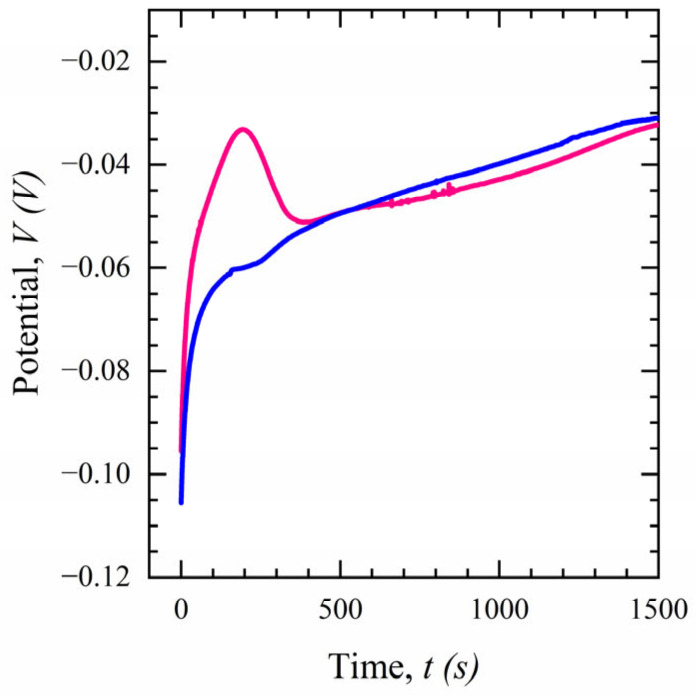
OCP profiles of two functionalized sensors in 0.1 M PBS.

To analyze the equilibrium time, the open circuit potential (OCP) for the fully modified electrode in PBS (0.1 M) is measured to find the time to thermodynamic equilibrium for the sensor. Figure 9 shows the OCP result of two modified sensors using PBS as the electrolytic control solution with a duration of 1500 s. As can be seen, a significant difference in the OCP is observed at t < 300 s, resulting in a large variance in the output of the device, which is eminently undesirable. However, the variance decreases as the OCPs converge at t > 300 s, indicating that the sensors’ response becomes stable and reaches an equilibrium. As the rapidity of detection is one of the main concerns for POC devices, a minimum time of 5 min is chosen for the incubation time.

### 4.3. Sensitivity, Selectivity, and Stability of the Biosensor

**Figure 10:**
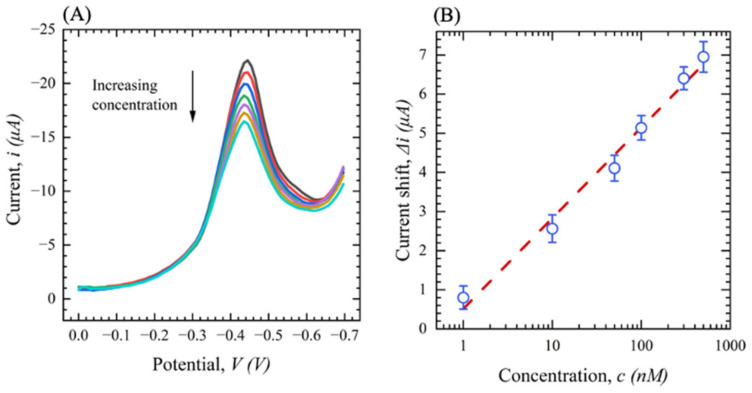
(**A**) SWV for functionalized sensors with different concentrations of S protein from (0–500 nM) in 0.1 M PBS (*n* = 3); (**B**) current shift (∆*i*) as a function of S protein concentration in 0 M PBS (*n* = 3).

The final characterizations of the sensor developed in this work are performed under optimal conditions (pH = 8 and 5 min incubation) and performed via SWV. Figure 10 shows the SWV response of devices in the S protein with concentrations ranging from 0 (0.1 M PBS) to 500 nM voltammetry at a frequency of 20 Hz, a square wave amplitude of 0.05 V, a potential range of −0.2 to −0.7 V, and a potential step of 5 mV. As concentrations increase, the peak current decreases, indicating that Ab-Ag binding produces changes in the Th reaction on the surface (Figure 10A). Figure 10B demonstrates the observed ∆*i* with respect to the S protein concentration. The sensor shows an excellent linear correlation between ∆*i* and the S protein concentration with R^2^ of 0.97 and RSD < 4%.

To rigorously analyze the response of the sensor, the LOD is calculated using the equation *LOD =* 3*N/SE*, where *N* is the noise of the sensor response in a blank solution, and *SE* is the sensitivity of the sensor. It is obtained that the LOD of the sensor for detecting the S protein is 200 pM.

**Figure 11:**
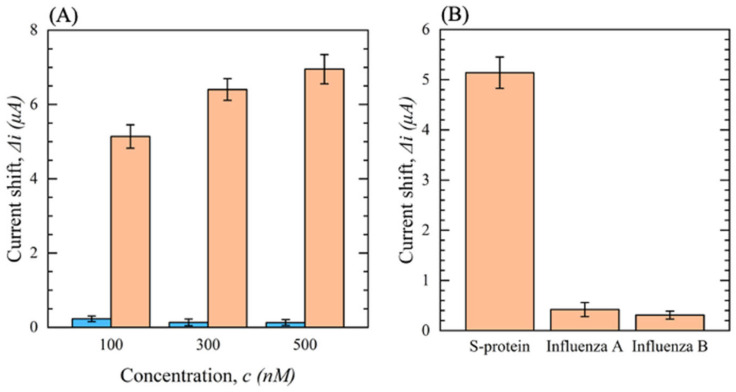
(**A**) Current shift (∆*i*) of functionalized sensors with S1-Ab (orange) and mouse IgG (blue) as a function of S protein concentration in 0.1 M PBS (*n* = 3); (**B**) the cross-reactivity response of the biosensor comparing 100 nM of S protein and 100 nM of influenza A and B antigens (HA) (*n* = 3).

To assess the selectivity of the biosensor and ensure that the change in current seen is due to surface binding and not the settling of proteins on the surface, various negative control tests were conducted. Mouse IgG was immobilized on the sensor in the place of the S1-Ab used on the normal functionalized sensors. The signal was measured after dropping the S protein in PBS with concentrations ranging from 0 to 500 nM. The results are shown in Figure 11A. As the concentration increases, the sensor with immobilized mouse IgG Ab shows no significant change in ∆*i*, whereas a significant increase is obtained for the S1-Ab. This confirms that the Ab-Ag binding is the primary source of the increase in ∆*i*. Furthermore, it is essential to investigate the cross-reactivity of the developed biosensor to other viruses with similar symptoms to SARS-CoV-2 such as influenza A and B. In total, 100 nM samples of the S protein, hemagglutinin (HA) protein of influenza A, and HA of influenza B were dropped on the biosensor, and the current shift, with respect to a control solution, was measured. The results are shown in Figure 11B. A significant current shift is seen for the sample containing the S protein, whereas the influenza A and B antigens show negligible shifts. The results indicate that the developed biosensor shows outstanding selectivity for SARS-CoV-2 detection.

**Figure 12:**
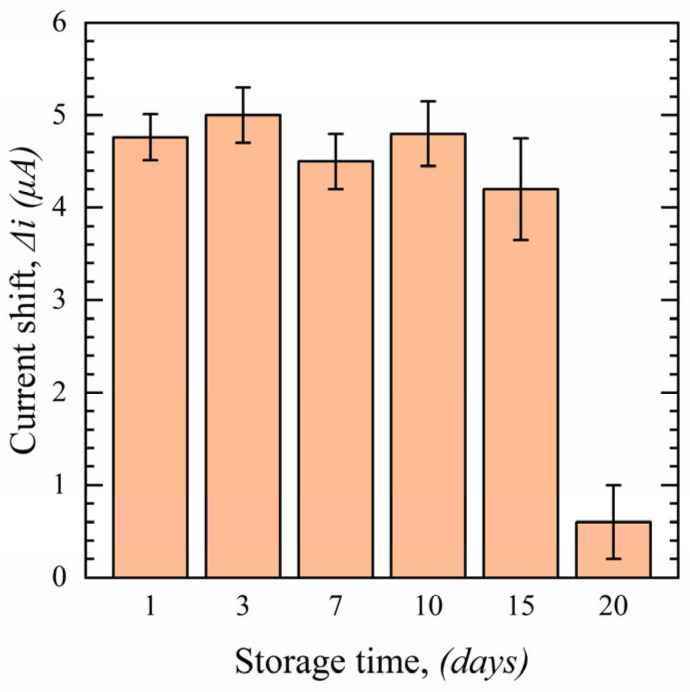
Stability of the functionalized sensors over 20 days in 100 nM S protein in 0.1 M PBS (*n* = 3).

To examine the stability of the sensor, experiments were conducted using 100 nM of SARS-CoV-2 S protein in PBS over 20 days, and the results are shown in Figure 12. As can be seen, sensors can be preserved in vacuum-sealed packages at 4 °C for up to 15 days without compromising their initial performance. However, a significant performance decline is observed after 20 days due to S1-Ab degradation.

### 4.4. Salivary Spike Protein Detection

**Figure 13:**
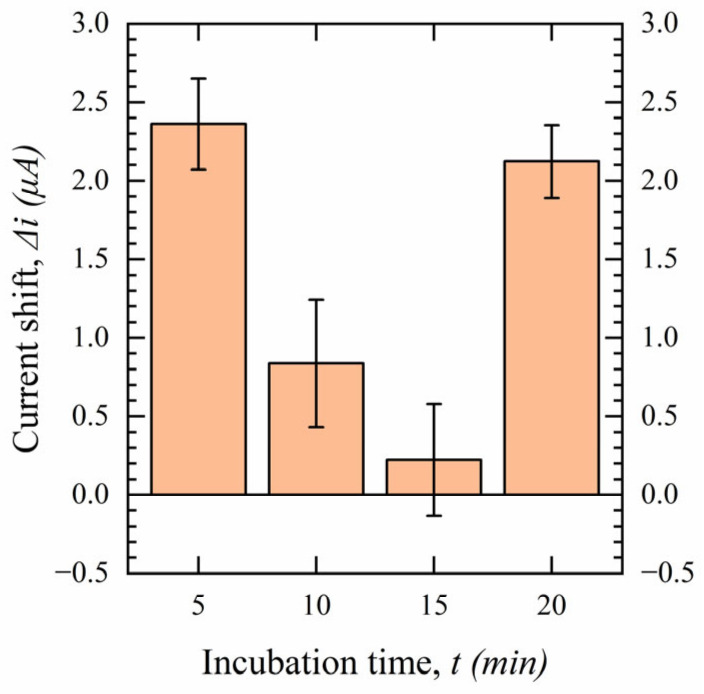
Effect of incubation time on sensor performance in subject’s filtered saliva (*n* = 3).

To evaluate the performance of the biosensor more rigorously, it is necessary to use saliva as a medium, as saliva contains thousands of proteins and other biomolecules that can affect the measurement. The saliva of one subject was collected through our sampling procedure and filtered with our previously stated process. Using saliva instead of PBS requires additional optimization of some of the testing parameters as the proteins and other biomolecules present in the sample can increase the diffusion time of the target molecule to the WE double layer to bind to the bioreceptor, and interfering molecules may land on the surface blocking binding sites. Most importantly, the incubation time needed to be re-examined. To test this, experiments were conducted with filtered saliva samples containing 100 nM of S protein with an incubation time ranging from 5 to 20 min., with the results shown in Figure 13.

The incubation time of 5 min. gives an increase in the current shift of the SWV in the presence of the S protein in saliva. This result shows a reduction in the target diffusion time in filtered saliva by removing most of the interferences and complexities of the solution through filtration. If the incubation time is increased to 10 or 15 min., the Δi is decreased, which is caused by the dissociation of the Ab-Ag bonds [55,56]. At an incubation time of 20 min., the Δi increased again where it is possible that the antigen reattaches to the binding sites. As it gives the highest sensitivity, and a low incubation time is eminently desirable for POC and OTC devices, an incubation time of 5 min. is chosen for salivary samples.

**Figure 14:**
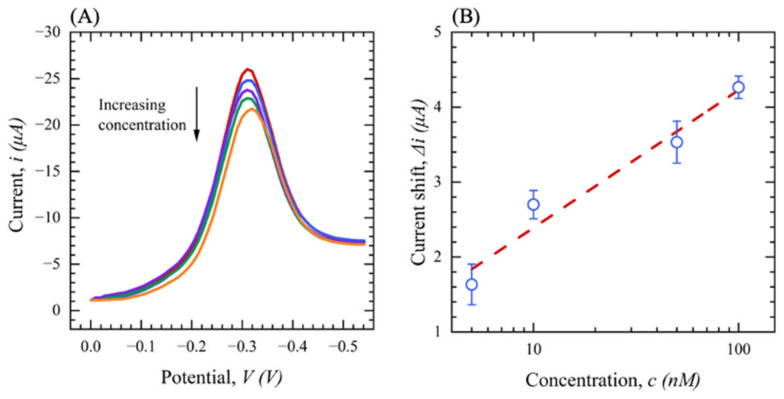
(**A**) SWV for functionalized sensors with different concentrations of SARS-CoV-2 S protein (0–100 nM) in the subject’s filtered saliva (red: filtered saliva (blank), blue: 5 nM, purple: 10 nM, green: 50 nM, orange: 100 nM (*n* = 3). (**B**) Current shift (∆*i*) as a function of SARS-CoV-2 S protein in subject’s filtered saliva.

Figure 14 shows the SWV of filtered saliva containing the S protein with concentrations ranging from 0 to 100 nM. Here the potential range was 0 to −0.7 V, and the square wave frequency was 20 Hz. As the concentration increases, the SWV peak current decreases, indicating that Ab-Ag binding produces changes in thionine’s reaction kinetics over the surface, as we discussed in Section 2 (Figure 14A). Figure 14B shows the obtained ∆*i* with respect to the S protein concentration. The sensor shows a reliable linear range of detection between 5 to 100 nM with an R^2^ of 0.93 and RSD < 3%. The sensor has a LOD of 500 pM for the S protein in filtered saliva which is slightly higher than the S protein in PBS (200 pM) due to the different and complex matrix of saliva compared with the PBS solution. However, although the person-to-person variation in the composition of saliva is high (proteins, electrolyte concentration, pH, etc.), these results show promising potential for the rapid detection of antigens in saliva using our electrochemical sensors and single step filtration process.

### 4.5. COVID-19 Pseudovirus Detection

In the previous section, it is shown that the developed sensor is able to detect the S protein in PBS with a LOD of 200 pM. However, the detection of whole virions in real samples comes with more complications. The mass of SARS-CoV-2 virions is around 10^4^ times greater than its S protein, and there are around 90 S proteins per particle of the virus [57]. Positive samples (NP or saliva) can only be handled in a BSL-4 laboratory, so to test the sensor with whole virions, a pseudovirus is used, which is a lentivirus modified to express the SARS-CoV-2 S protein on its surface.

**Figure 15:**
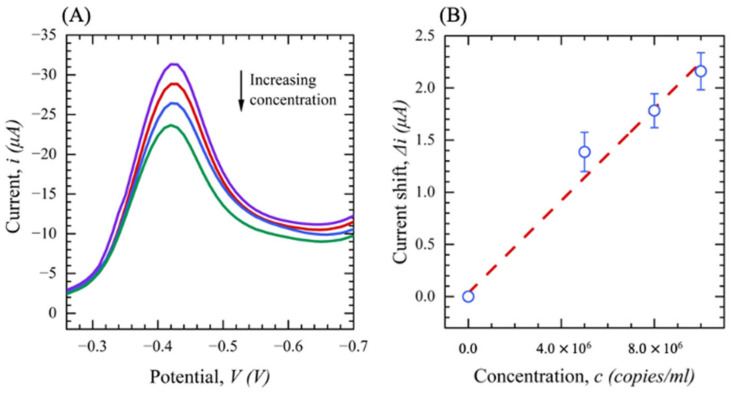
(**A**) SWV of SARS-CoV-2 pseudovirus; (purple: DMEM + FBS (no PV), red: 5 × 10^6^, blue: 8 × 10^6^ green: 10^7^ copies/mL). (**B**) The current shift (∆*i*) as a function of PV concentration in DMEM+10 %FBS (*n* = 3).

The SARS-CoV-2 pseudovirus (PV) with a concentration of 1 × 10^7^ copies/mL was purchased from My BioSource and was stored in DMEM + 10% FBS. Different concentrations of PV solutions were prepared from 0 to 1 × 10^7^ copies/mL by diluting the stock solution. Then, the functionalized sensors were inserted into the device, where the sample is applied to the sensor functional area and incubated for 5 min. After incubation time, SWV was performed with a potential range of 0 to −0.7 V and a frequency of 20 Hz. A control experiment was conducted with DMEM + 10% FBS to establish the baseline, and the measurements were repeated for the solutions containing a range of concentrations of the PV. The viral load concentration was then correlated to the shift in the current from the baseline. Figure 15A shows the SWV response of the sensor in DMEM + 10% FBS solution with PV concentration ranging from 0 to 1 × 10^7^ copies/mL. As the viral load increases, the peak currents are reduced due to the attachment of the virus on the electrode surface changing the properties of the chemistry and occluding some of the thionine’s electron transfer pathway. Figure 15B represents the observed current shift (∆*i*) with respect to different concentrations of PV. The results show good linearity down to a concentration of 5 × 10^6^ copies/mL with a sensitivity of 2.004 × 10^−7^ µA /(copies/mL)^−1^ and LOD of 10^6^ copies/mL.

**Figure 16:**
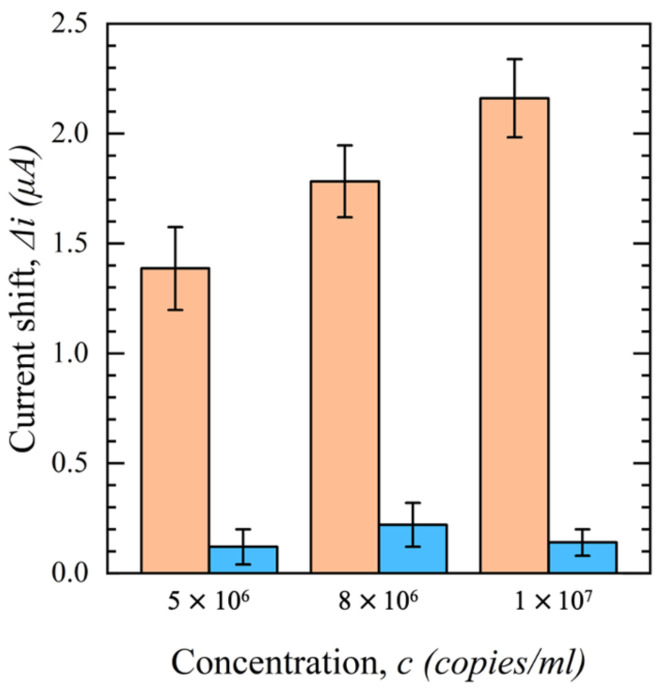
Current shift (∆*i*) of functionalized sensors with S1-Ab (orange) and mouse IgG (blue) as a function of PV concentration in DMEM + 10% FBS (*n* = 3).

As with the other testing conditions performed earlier in this work, a negative control test was performed with mouse IgG as the immobilized bioreceptor. The same procedure to assess the selectivity of the biosensor was performed, and the results are shown in Figure 16. A significant shift in the output current (∆*i*) with an increasing concentration of PV was observed when S1-Ab was used. However, no significant change was recorded when the mouse IgG was immobilized on the modified sensors. Based on the obtained results, one can conclude that the change in ∆*i* is due to the specific binding of PV to S1-Ab, not virions settled on the surface through non-specific physical adsorption.

## 5. Conclusions

The successful implementation of the CNT/GNp and Th/S1-Ab LBL assembly as a suitable surface for the label-free electrochemical detection of SARS-CoV-2 on a Pt sensor is introduced in this work. Th, as a redox probe, is embedded in the functional chemistry precluding the need for additional redox probes or tags into the sample solution as with prevalent SARS-CoV-2 diagnostic techniques used today. Moreover, this disposable electrochemical biosensor demonstrates high sensitivity and selectivity for detecting SARS-CoV-2 S protein and a SARS-CoV-2 pseudovirus in 5 min. The sensor has a limit of detection (LOD) of 200 and 500 pM for freely suspended S protein in PBS and human saliva, respectively. In addition, the sensor’s performance was proven for detecting a COVID-19 PV in DMEM + 10% FBS with a sensitivity of 2.004 × 10^−7^ µA /(copies/mL)^−1^ and a LOD of 10^6^ copies/mL. Moreover, the sensor shows stability over 15 days of storage and good repeatability over triplicate tests with an RSD < 4%. The developed sensor is also a potential monitoring device for disease progression in infected users as a quantitative sensor will show if the viral load is not falling over time, and hospitalization is needed. The electrochemical technique described in this work represents a step forward toward a viable platform for point-of-care and at-home testing that has an advantage over traditional qualitative methods.

## 6. Future Work

Future work will be initiated on (i) conducting a clinical trial to investigate the sensor’s performance on individuals’ samples, (ii) improving the shelf life of the sensor for scaled distribution, and (iii) modifying the sensor by switching out the bioreceptor for use with other infectious diseases or chronic conditions.

## 7. Patent

Rapid Electrochemical Point-of-Care COVID-19 Detection in Human Saliva US 17/351,211 Issued 11 September 2021.

## Data Availability

All data have been reported in the manuscript.
